# A novel Turkish instrument for assessing quality of life in chronic otitis media –translation and validation of Zurich chronic middle ear inventory

**DOI:** 10.3906/sag-2003-38

**Published:** 2020-12-17

**Authors:** Belgin TUTAR, Ziya SALTÜRK, Güler BERKİTEN, Muhammed Enis EKİNCİOĞLU, Semih KARAKETİR, Ece ARKAN, Muhammed Fatih AKGÜN, Ayça Başkadem YILMAZER, Ercan KULAK, David BӒCHİNGER, Yavuz UYAR

**Affiliations:** 1 Department of Otorhinolaryngology-Head and Neck Surgery, Okmeydanı Training and Research Hospital, İstanbul Turkey; 2 Department of Public Health , Marmara University, İstanbul Turkey; 3 Department of Otorhinolaryngology, Head and Neck Surgery, University Hospital Zurich, Zurich Switzerland

**Keywords:** ZCMEI-21, chronic otitis media, quality of life, Turkish

## Abstract

**Background/aim:**

The Zurich Chronic Middle Ear Questionnaire (ZCMEI-21) is a newly-developed German-language questionnaire. The purpose of this study was to analyze the quality of life (QoL) of chronic otitis media (COM) patients and translate, transculturally adapt, and validate the ZCMEI-21 into Turkish.

**Materials and methods:**

Based on internationally accepted guidelines, the ZCMEI-21 was translated into Turkish. To assess its validity, the total score of the ZCMEI-21-Tur was compared to the scores taken from the original validation study and a question that was directly related to the health-related QoL (HRQoL), as well as the general criterion EQ-5D-5L. Questionnaires were completed by healthy volunteers and the results were evaluated statistically.

**Results:**

A total of 80 COM patients and 40 healthy volunteers were prospectively enrolled in this study. Regarding internal consistency, the questionnaire showed a Cronbach α of 0.94, which indicated high internal consistency. Moreover, internal consistency was also determined to be excellent for the Cronbach α of the individual subscales, as follows: ear sign symptoms, 0.79; hearing, 0.83; psychosocial impact, 0.91, and medical resources, 0.84.

**Conclusions:**

The ZCMEI-21 was translated into Turkish and validated. Therefore, the ZCMEI-21-Tur was suitable for use in assessing HRQoL in adult patients with COM.

## 1. Introduction

Chronic otitis media (COM) affects approximately 2% of the population. Otorrhea, hearing loss, tinnitus, vertigo, and malodorous discharge are serious problems in patients with COM. In particular, hearing loss and odor-related problems may cause problems for the patient when communicating with their environment and consequently, problems in education, and professional and social life [1]. Like many other chronic diseases, COM is a triggering factor for chronic stress and psychological problems. The parameters used for evaluation in diagnosis and the choice of treatment are related to the disease, but do not reflect the personal experiences of the patients. Questionnaires that assess the patients subjectively are needed to assess their challenges and motivation for treatment [2].

The Zurich Chronic Middle Ear Questionnaire (ZCMEI-21) is a newly-developed German-language questionnaire that comprehensively evaluates the symptoms and psychosocial effects of health-related quality of life (HRQoL) in COM patients [3]. The English, German, and Italian versions were found to be valid and consistent; however, English, German, and Italian belong to the Indo-European language family. Validation of a Japanese language version was performed and found to be suitable [4–6]. Japanese is from the Ural-Altaic language family, like Turkish, and has a similar structure. However, the only ear disease-related questionnaire translated into Turkish was the chronic otitis media questionnaire-12 (COMQ-12), which only briefly mentions lifestyle effects and is heavily oriented around hearing-related impairment of HRQoL [7]. Therefore, there is a need for a measurement to evaluate patients more comprehensively. In this study, the aim was to translate the ZCMEI-21 into Turkish and evaluate its cross-cultural adaption and validation in order to assess the quality of life (QoL) of patients with COM.

## 2. Material and methods

### 2.1. Patients

Ethical permission approval was obtained from the Ethics Committee of our institution under approval number 48670771-000-8112. Informed consent form was given by all of the participants.

### 2.2. Inclusion criteria

Preoperative and postoperative patients with preoperative or postoperative COM simplex (OMCS) or COM with cholesteatoma (OMCC) who were older than 18 years old were invited to participate in the study. The control group included patients with no ear complaints.

### 2.3. Translation and cross-cultural adaptation

**Figure 1:**
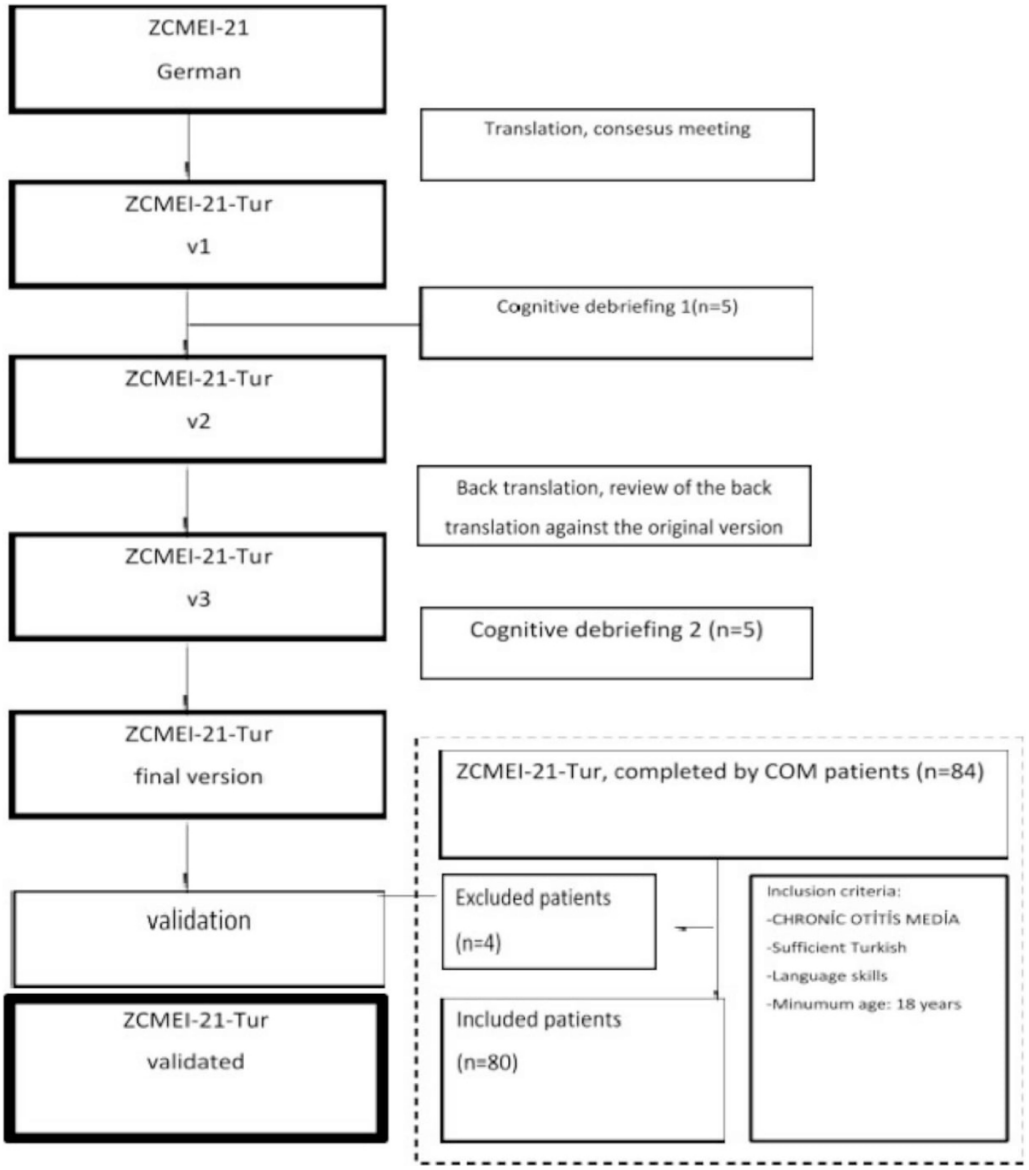
Flow chart of the translation and validation approach.

Translation from German to Turkish was completed by 2 professional translators. These 2 inventories were then combined into a single file by physicians and translators (ZCMEI-21-Tur, v1). This first version was applied to 5 patients. The problems that the patients had in understanding the questions were evaluated, and minor changes were made (ZCMEI21-Tur, v2). A third professional translation agent then did a German translation of the final version, and the ZCMEI-21-Tur was changed into a conceptually equalized translation (ZCMEI-21-Tur, v3). The second pilot test (cognitive questioning 2) was performed with another 5 COM patients. No changes were made. The final version of the questionnaire (ZCMEI-21-Tur) was applied to both groups of patients and healthy volunteer subjects (Figure 1).

### 2.4. Validation

The original German questionnaire was administered to patients via an electronic version. The patients were interviewed face-to-face and administered the questionnaire in person. The questionnaires (ZCMEI-21-Tur and EQ-5D-5L) were administered by a single resident to 84 patients who met the inclusion criteria and to 40 healthy volunteers. Of the patients, 4 were excluded from the study due to incomplete questionnaires. Question 22, which was in the original validation study, was included in the questionnaire. This was a general question that addressed HRQoL directly: “The disease in my ear worsens my quality of life”.

The EQ-5D-5L version consists of 5 main questions, and each question has 5 answers. The 5 questions cover the mobility of the patient, self-care, daily activities, and pain/discomfort related to COM, and anxiety/depression caused by disease [8]. Patients choose from 1- to 5-point Likert-type appropriate responses. It also includes a visual analog scale (VAS) score. This VAS is based on describing their health status on a scale from 0 to 100, with 100 corresponding to the best health condition. The survey was completed by 120 people and the survey results were evaluated statistically.

### 2.5. Statistical analysis

Values were reported as the mean ± SD or as an absolute number and percentage. The item total-correlation was calculated in order to evaluate whether it correlated with the total score. An item total-correlation of ≥0.3 indicated that a question correlated well with the total score and represented 1 criterion for a strong item. Construct validity was established using a question (question 22) that directly assessed HRQoL. For assessing the reliability of the ZCMEI-21-Tur, internal consistency, as an indicator of reliability, was determined using the Cronbach α. Concurrent validity was determined by comparing the ZCMEI-21-Tur total scores and subscores to the EQ-5D descriptive system and VAS scores using the Spearman rank correlation and linear regression analysis, including mean prediction intervals. Statistical significance was accepted as P < 0.05.

## 3. Results

A total of 80 patients and 40 controls completed the ZCMEI-21-Tur and EQ-5D-5L. Thus, a total of 120 participants, with a mean age of 30.4 ± 9.2 (between 18 and 45) years in the validation group and 29.9 ± 8.9 (18–44) years in the control group, were included in the study. OMCC was the diagnosis in 29 (36%) patients and OMCS was seen in 51 (64%) patients. At the time of fulfillment of the ZCMEI-21-E, 62 (78%) patients had already undergone surgery for COM. Additionally, 40 had undergone tympanoplasty and 22 had tympanomastoidectomy. The air conduction threshold of the patients was 46.3 ± 8.2 dB HL, and the bone conduction threshold was 26.6 ± 6.9 dB HL. The air conduction threshold of the control group was 15.6 ± 6.1 db HL, while the bone conduction threshold was 9.5 ± 4.5 dB HL. Detailed characteristics of the patients and control group are given in Table 1.

**Table 1:** Patient characteristics of the study population and control group.

	Validation group(n = 80)	Controls(n = 40)
Male to female ratio	38:42	19:21
Age (mean ± SD) (years)	30.36 ± 9.22	29.87 ± 8.93
COM, type—no. (%)		N/A
OMCC	29 (36 %)	
OMCS	51 (64 %)	
Affected ear(s)—no. (%)		N/A
Right	48 (60 %)	
Left	29 (36 %)	
Both	3 (4 %)	
Previous operation due to COM—no. (%)	62 (78 %)	N/A

COM, chronic otitis media; OMCS, otitis media chronica simplex; OMCC, otitis media chronica cholesteatomatosa.

Concerning the single-item descriptive statistics of the ZCMEI-21-Tur, it was found that the answers were well distributed and the full range of answers was used on every question. Item-total correlations of all of the items were well above 0.3 (for detailed descriptive statistics, see Table 2). An item-total correlation of ≥0.3 indicated that an item correlated well with the total score, representing 1 criterion for a strong item. Assessing the internal consistency as an indicator of reliability, the questionnaire showed a Cronbach α of 0.94, indicating high internal consistency. Adequate to excellent internal consistency was also found for the individual subscales (Table 2).

**Table 2:** Descriptive statistics of the individual items (ZCMEI-21-Turkish).

	Mean	SD	Min–max	ITC	Cronbachα
I. Ear signs and symptoms					0.79
1. Ear pain	1.00	0.81	0–3	0.66	
2. Discharge	0.65	0.81	0–3	0.75	
3. Itching	0.90	1.00	0–4	0.42	
4. Feeling of pressure	0.84	0.74	0–2	0.60	
5. Balance	0.43	0.63	0–2	0.48	
II. Hearing					0.83
6. Tinnitus	1.46	1.42	0–4	0.48	
7. Hearing (filter question)	1.84	1.01	0–4	0.67	
8. When many people speak at the same time	1.65	1.06	0–4	0.70	
9. Telephone, alarm clock	1.81	1.26	0–4	0.65	
10. Fear of not hearing other people	1.53	1.12	0–4	0.64	
III. Psychosocial impact					0.91
11. Impact of ear symptoms on HRQoL	1.96	1.35	0–4	0.81	
12. Protection from water	1.00	1.30	0–4	0.55	
13. Activities with family and friends	1.78	1.34	0–4	0.74	
14. In public (e.g., school/occupation, shopping)	1.35	1.03	0–4	0.67	
15. Making contact with other people	1.60	1.34	0–4	0.79	
16. Quality of sleep	2.89	1.38	0–4	0.79	
17. Sadness	2.85	1.18	0–4	0.71	
18. Fear that the ear problems may persist	1.73	1.11	0–4	0.63	
IV. Medical resources					0.84
19. Medical consultations	1.68	0.99	0–4	0.71	
20. Antibiotics (oral)	1.26	1.06	0–3	0.75	
21. Ear drops	1.75	1.17	0–4	0.64	

ITC: item total correlation; min: lowest value; max: highest value; α: Cronbach α for the subscales.

**Figure 2:**
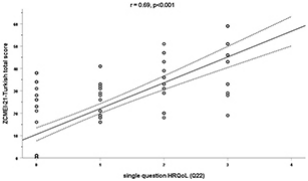
Spearman’s rank correlation of the ZCMEI-21-Turkish total scores and the question directly addressing HRQoL (0 on the x-axis corresponds to no impact on HRQoL, as indicated by the question directly addressing HRQoL, whereas 4 indicates the greatest impact; Spearman’s rank correlation.

**Figure 3:**
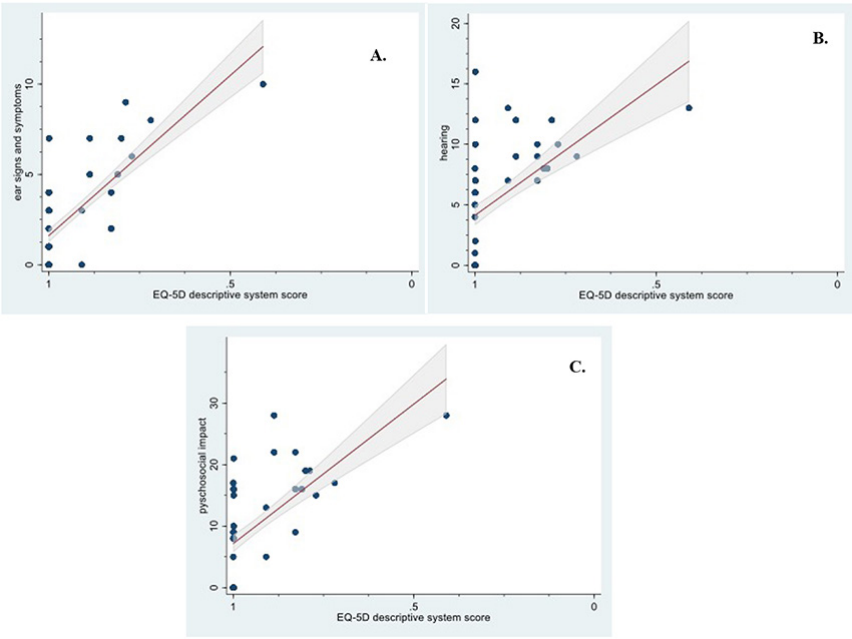
(A–C) Spearman’s rank correlation between the ZCMEI-21-Turkish subscale scores and EQ-5D descriptive systems score. Solid line represents linear regression, dashed lines represent 95% prediction intervals. A) r = 0.71, P < 0.001. B) r = 0.69, P < 0.001. C) r = 0.65, P < 0.001.

**Figure 4:**
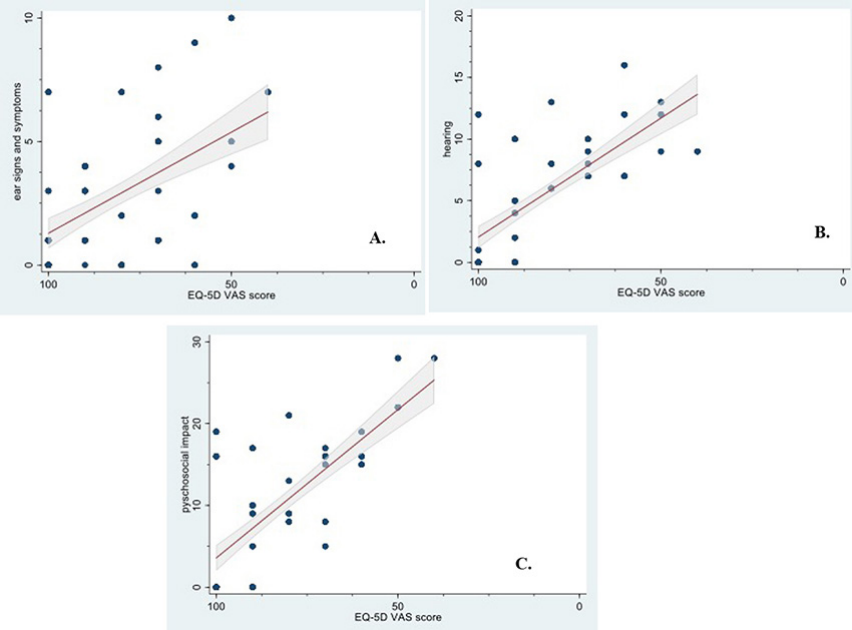
(A–C) Spearman’s rank correlation between the ZCMEI-21-Turkish subscale scores and EQ-5D VAS score. Solid line represents linear regression, dashed lines represent 95% prediction intervals. A) r = 0.48, P < 0.001. B) r = 0.68, P < 0.001. C) r = 0.64, P < 0.001.

When comparing the total score and direct HRQoL question, a strong correlation (r = 0.69, P < 0.001) was found. When comparing the ZCMEI-21-Tur total score and EQ-5D scores, strong correlations (r = 0.76, P < 0.001 for the descriptive system score and r = 0.70, P < 0.001 for the VAS score) were found. The total score of the ZCMEI-21-Tur and the scores of its subscales, comprising ear signs and symptoms (questions 1–5), hearing (questions 6–10), psychosocial impact (questions 11–18), and medical resources (questions 19–21) are given in Table 3 and Figures 2, 3A–3C, and 4A–4C).

**Table 3:** Comparison and correlation of the total scores and subscales scores of the ZCMEI-21 and ZCMEI-21-Turkish and EQ-5D-5L.

	Current study ZCMEI-21-Turkish (n = 80)	Original validation study ZCMEI-21 (n = 76)
ZCMEI-21-Turkish/ZCMEI-21 total score (±SD)	31.9 ± 10.9	29.7 ± 16.1
I. Ear signs and symptoms	3.8 ± 2.8	5.1 ± 3.9
II. Hearing	8.3 ± 3.4	8.5 ± 5.2
III. Psychosocial impact	15.2 ± 6.1	13.1 ± 7.9
IV. Medical resources	4.7 ± 2.5	3.0 ± 2.3
EQ-5D-5L (±SD)		
Descriptive system score	0.90 ± 0.13	0.92 ± 0.14
VAS score	74.8 ± 16.8	N/A
Total score correlation*		
To question directly assessing HRQoL (Q22)	r = 0.69, P ˂ 0.001	r = 0.74, P ˂ 0.001
To EQ-5D-5L descriptive system score	r = 0.76, P ˂ 0.001	r = 0.60, P ˂ 0.001
To EQ-5D VAS score	r = 0.70, P ˂ 0.001	N/A

*Spearman’s rank correlation coefficient, P-value.

Because a significant portion of the validation group had already undergone surgical treatment for COM (78%), the differences between the ZCMEI-21-Tur total scores of the patients who had undergone surgery and those who had not undergone surgery were assessed. The mean ZCMEI-21-Tur score patients who had undergone surgery was 29.0 ± 8.4 and for those who had not undergone surgery it was 41.9 ± 12.8 (P < 0.001). The results of the comparison of the ZCMEI-21-Tur scores between the patients with COM and the control group are given in Table 4. The patients had higher mean total scores and SDs when compared to the control group (P < 0.001).

**Table 4:** Comparison of the ZCMEI-21-Turkish scores between patients with COM and healthy participants serving as the controls (mean ± SD, Mann–Whitney U test).

	COM (validation group)	Controls	P-value
I. Ear signs and symptoms	3.8 ± 2.8	0.7 ± 0.5	˂0.001
II. Hearing	8.3 ± 3.4	0.0 ± 0.0	˂0.001
III. Psychosocial impact	15.2 ± 6.1	0.0 ± 0.0	˂0.001
IV. Medical resources	4.7 ± 2.5	0.0 ± 0.0	˂0.001
Total score	31.9 ± 10.9	0.7 ± 0.5	˂0.001

## 4. Discussion

In recent years, patient-reported outcome measures (PROMs) have become of great importance for follow-up, evaluation, and research [9]. There are 3 commonly used questionnaires for PROMs in adult patients with COM, comprising the chronic ear survey (CES, English), Chronic Otitis Media Outcome Test-15 (COMOT-15, German), and COMQ-12 (English) [10]. Each survey highlights a different area. In this study, the ZCMEI-21 was successfully translated into Turkish and validated.

The CES was developed by Nadol et al. in 2000 and contains 13 questions on 3 main topics: activity restriction, symptoms, and medical resources. It does not include questions about tinnitus or dizziness, and it also does not evaluate the psychology or decrease in QoL of the patient [1,10]. The COMOT-15 was introduced by Baumann et al. in 2009. It includes questions about ear symptoms, hearing, and mental status. It does not inquire about anxiety, depression, or social isolation, which have specific importance in COM [11,12]. The COMQ-12 questionnaire was developed in 2014 by Phillips et al. It contains 12 questions in 4 categories, comprising symptoms, lifestyle and work, healthcare, and general impact. Although it contains questions about impact on lifestyle and work, it does not include questions that meet the psychological and social dimensions of COM [13].

In 2016, Bächinger et al. [3] developed a new German electronic questionnaire, ZCMEI-21, for the comprehensive measurement of HRQoL, which aimed to evaluate adult COM patients in all aspects of the disease and its consequences on HRQoL. Although the ZCMEI-21 is based on current surveys (CES, COMOT-15), it varies in several aspects that provide certain advantages. The ZCMEI-21 evaluates classic ear symptoms (e.g., pain, discharge, dizziness/balance problems, hearing impairment, and tinnitus). The pain-related element is particularly important concerning patients who have recently undergone surgery. The ZCMEI-21 is classified into 4 subheadings: I) ear signs and symptoms, II) hearing, III) psychosocial effects, and IV) medical resources. Regarding the psychological and social aspects of the patients, the ZCMEI-21 is valuable in the evaluation of neglected aspects of the disease. Baechinger et al. administered the ZCMEI-21 to 76 patients with COM. They compared the total score of the ZCMEI-21 with a question that directly reflected HRQoL and the total score of the EQ-5D questionnaire. The ZCMEI-21 total score and EQ-5D descriptive system score were only moderately correlated (r = 0.60, P < 0.0001), while the ZCMEI-21 total score and the question directly related to HRQoL showed a strong correlation (r = 0.74, P < 0.0001). Verification showed a Cronbach α of 0.91 and excellent internal consistency. In addition, the ZCMEI-21 was able to differentiate patients with COM from healthy participants (P < 0.0001). Thus, good sensitivity was obtained. As a result, sufficient information was obtained about reliability and validity to extend the application of the ZCMEI-21 to quantify HRQoL in patients with COM.

The current study of the ZCMEI-21-Tur revealed a Cronbach α of 0.94, indicating higher internal consistency than the Japanese and English versions. Adequate to excellent internal consistency was also found for the individual subscales. When comparing the ZCMEI-21-Tur total score and the question directly assessing HRQoL, a strong correlation (r = 0.69, P < 0.001) was detected. When comparing the ZCMEI-21-Tur total score and the EQ-5D scores, strong correlations were obtained (r = 0.76, P < 0.001 for the descriptive system score and r = 0.70, P < 0.001 for the VAS score).

There were some limitations of the study, including the limited number of participants and heterogeneous COM group. Both preoperative and postoperative patients were included the study, which may have seemed confusing, but the original paper, English, and Japanese validations were designed to include both; hence the current study did as well. On the other hand, the total number of participants was sufficient to perform the statistical analysis.

**Conclusion:** The ZCMEI-21-Tur is suitable for use in assessing HRQoL in adult patients with COM. It may therefore be used for following-up patients for research purposes, as well as in clinical practice.
